# Editorial: Drug Repurposing for COVID-19 Therapy

**DOI:** 10.3389/fphar.2021.748886

**Published:** 2021-08-24

**Authors:** Rafael Maldonado, Filippo Drago

**Affiliations:** ^1^ Barcelona Biomedical Research Park (PRBB), University Pompeu Fabra, Barcelona, Spain; ^2^ IMIM (Hospital Del Mar Medical Research Institute), Barcelona, Spain; ^3^ Clinical Pharmacology Unit/Regional Pharmacovigilance Centre, University Hospital of Catania, Catania, Italy; ^4^ Department of Biomedical and Biotechnological Sciences, University of Catania, Catania, Italy; ^5^ Centre for Research and Consultancy in HTA and Drug Regulatory Affairs (CERD), University of Catania, Catania, Italy

**Keywords:** COVID-19, repurposing, emergency, drug reuse, pandemic

The rapid emergence in December 2019 of cases of severe acute respiratory syndrome coronavirus 2 (SARS-CoV-2) infection in China rapidly expanded to multiple countries leading to a pandemic situation in March 2020 and dramatic changes worldwide. COVID-19 immediately had major health consequences due to its severity, mainly in the population at risk, and to the lack of effective treatment to ameliorate the prognosis of the disease. Indeed, SARS-CoV-2 infection causes respiratory symptoms that range from mild forms to more serious ones, causing pneumonia, and multi–organ damage. Moreover, the sudden appearance and rapid propagation of COVID-19 produced an unexpected socio-economic crisis and major efforts have been devoted by multiple professionals to try to minimize the burden generated by this disease.

From the beginning of the pandemic, the scientific community made enormous efforts in order to rapidly develop vaccines that prevent the propagation of the SARS-CoV-2 infection. These research efforts result into an unprecedented success by reaching to the development of several efficient and secure vaccines in a time record in the history of vaccine development. Standard adenoviral approaches and novel mRNA strategies were used to successfully develop these novel vaccines and now there are still two enormous challenges opened for reaching an efficient vaccination campaign: the rapid distribution of these vaccines worldwide and the needs to raise awareness in the population about the safety and essential requirement of these vaccines to fight the COVID-19 pandemic.

Simultaneously to the vaccine development, multiple scientific groups concentrate their activities in an attempt to identify effective and safe pharmacological treatments against COVID-19. Indeed, both vaccines and pharmacological treatments are complementary to avoid the transmission of the viral infection and to prevent the severe consequences of the disease. In spite of the progress in the vaccination campaigns, pharmacological interventions are still needed to treat patients suffering the disease and to palliate the long-term consequences of the persistent forms of COVID-19. The efforts of the research were mainly devoted to the identification of compounds with anti-SARS-CoV-2 activity as well as drugs able to minimize the dramatic consequences of the exaggerated immune response leading to the most severe forms of the disease. However, due to the urgent need for a rapid development of pharmacological strategies, there was no time to start the long process required to develop novel compounds for such purposes. Therefore, the dominant research strategy was repurposing drugs for COVID-19 that were previously developed for other therapeutic purposes.

Research efforts of the scientific community were quickly translated in a large number of publications, including those devoted to the development of pharmacological approaches. The large majority of these publications met the rigorous criteria required for any prestigious scientific article. However, some few exceptions led to sound retractions that were largely commented and discussed by the general media, which emphasized once again the needs of the well-known rigorous peer review process in any scientific publication.

In order to collect the best evidence about drugs repurposed for COVID-19, we proposed and coordinated since May 2020 this Research Topic.

Several articles published in this Research Topic are devoted to antimalarial drugs that initially raised high expectancy due to their potential anti-SARS-CoV-2 activity. This initial interest was mainly focused on chloroquine and hydroxychloroquine, although the important risks associated to these treatments prompt overcome their potential benefits, as it is discussed and well-documented in several articles (Ren et al.; Kamat and Kumari; Manivannan et al.; Agarwal et al.; Younis et al.; Uckun et al.; Lozano-Cruz et al.). Antiretroviral drugs used for the Acquired Immunodeficiency Syndrome (AIDS) therapy, such as lopinavir and ritonavir, as well as antiviral drugs used for Ebola Viral Disease treatment, such as remdesivir, were also initially repurposed for COVID-19 therapy. However, the high expectancy for these drugs also promptly turned down (Gagliardini et al.; Li et al.), even if remdesivir is still one of the few drugs approved by regulatory authorities for treatment of patients with COVID-19. Other interesting approaches have also been proposed as novel potential therapeutic strategies with antiviral activity against SARS-CoV-2 including targeting the sigma one receptor with selective or non-selective ligands, such as the antipsychotic compounds (Stip et al.; Vela), modified ovalbumin (Liang et al.), methylene blue (Bojadzic et al.), *Bacillus* Calmette-Guérin vaccine (Patella et al.), vitamin D (Boulkrane et al.), vitamin C (Zhao et al.) and compounds that may inhibit the binding of the viral spike protein to ACE2 (Tsegay et al.).

It has been demonstrated that an exacerbated inflammatory and immunological response to SARS-CoV-2 induces the most severe cases of the disease. The excessive production of pro-inflammatory cytokines may lead to a cytokine storm syndrome that aggravates the respiratory distress. Several drugs have also been repurposed in order to mitigate the dramatic consequences of this cytokine storm syndrome. The efficient repurposing of a particularly potent glucocorticoid drug, dexamethasone, that has already well-demonstrated the efficacy for such a purpose is discussed in this Research Topic (Gozzo et al.). Several immunosuppressant and anti-rheumatic drugs (Rubsamen et al.; Soldevilla-Domenech et al.; Mary et al.; Cavalli et al.; Sarabia de Ardanaz et al.; Pala et al.), as well as modulators of estrogen receptor activity (Calderone et al.) and the statins (Vuorio et al.), have also been proposed as potential therapies for the severe COVID-19 cases associated to this cytokine storm. Due to the high prevalence of thromboembolic complications that often appear mainly in the severe forms of COVID-19, the use of anticoagulants including heparin has been proposed and the current evidence for addressing this novel approach is also discussed in this Research Topic (Gozzo et al.; Drago et al.).

Multiple other cellular and molecular pathways have also been suggested as additional possible targets for the repurposing of drugs for COVID-19 therapy, as discussed in other articles included in our topic (Hussman; Sarkar et al.; Zhang et al.; Blaess et al.; Al-Motawa et al.; Chen et al.; Khan et al.; Bezemer and Garssen; Sharma et al.; Zuo et al.; Xiong et al.; De Crescenzo et al.). The therapeutic perspectives in particular high risk populations, such as diabetic patients, have also been discussed in this topic (Sun et al.), as well as the new challenges open for the diagnosis and pharmacoepidemiological follow up of COVID-19 (Bianco et al.; Shoaib et al.; Powell et al.).

Finally, several articles highlighted how the repurposing process, as well as the approval of COVID-19 therapy in general, has represented an enormous regulatory challenge which forced the regulatory systems to rapidly adapt their rules to the pandemic (Gozzo et al.; Sultana et al.; Andrade et al.).

We believe that drug reuse has been an important attempt as an emergency strategy in a serious situation that could recur in the future. We cannot rule out that similar pandemics still threaten people as long as globalization affects all human activities. Therefore, we must consider the experience of drug reuse for COVID-19 as extremely helpful in enriching our experience in seeking therapeutic solutions when serious global health hazards occur.

